# Design analysis of an MPI human functional brain scanner

**DOI:** 10.18416/ijmpi.2017.1703008

**Published:** 2017-03-23

**Authors:** Erica E. Mason, Clarissa Z. Cooley, Stephen F. Cauley, Mark A. Griswold, Steven M. Conolly, Lawrence L. Wald

**Affiliations:** a MGH-HST A.A. Martinos Center for Biomedical Imaging, Dept. of Radiology, Massachusetts General Hospital, Charlestown, MA, USA; b Harvard-MIT Health Sciences and Technology, Cambridge, MA, USA; c Case Western Reserve University, OH, USA; d University of California, Berkeley, CA, USA; e Harvard Medical School, Boston, MA, USA

## Abstract

MPI’s high sensitivity makes it a promising modality for imaging brain function. Functional contrast is proposed based on blood SPION concentration changes due to Cerebral Blood Volume (CBV) increases during activation, a mechanism utilized in fMRI studies. MPI offers the potential for a direct and more sensitive measure of SPION concentration, and thus CBV, than fMRI. As such, fMPI could surpass fMRI in sensitivity, enhancing the scientific and clinical value of functional imaging. As human-sized MPI systems have not been attempted, we assess the technical challenges of scaling MPI from rodent to human brain. We use a full-system MPI simulator to test arbitrary hardware designs and encoding practices, and we examine tradeoffs imposed by constraints that arise when scaling to human size as well as safety constraints (PNS and central nervous system stimulation) not considered in animal scanners, thereby estimating spatial resolutions and sensitivities achievable with current technology. Using a projection FFL MPI system, we examine coil hardware options and their implications for sensitivity and spatial resolution. We estimate that an fMPI brain scanner is feasible, although with reduced sensitivity (20×) and spatial resolution (5×) compared to existing rodent systems. Nonetheless, it retains sufficient sensitivity and spatial resolution to make it an attractive future instrument for studying the human brain; additional technical innovations can result in further improvements.

## I. Introduction

Magnetic Particle Imaging (MPI) is a tracer-based imaging technology introduced in 2005 [1] that detects the concentration of injected superparamagnetic iron oxide nanoparticles (SPIONs) using their nonlinear magnetic response. Due to the strong SPION magnetic moment and the zero-background signal in the human body (prior to SPION injection), MPI is anticipated to have sensitivity improvements over MR detection of contrast agents and to provide a background-free image. Additionally, MPI measures of SPION concentration are more easily rendered quantitative and have different tradeoffs between spatial and temporal resolution.

To date, rodent-sized MPI scanners exist [2, 3], but MPI has not been scaled for use on humans. Several barriers exist to increasing the scanner size, and the effect of this scaling on spatial resolution and sensitivity are only in the initial stages of investigation [4, 5]. Here we present a design analysis of an MPI scanner aimed at the needs of functional brain imaging.

For functional neuroimaging, we anticipate using MPI to directly map Cerebral Blood Volume (CBV) changes that occur in response to brain activation. Activated areas of the brain are observed to have CBV changes of about 20%[6]. Since the cerebral SPION does not cross the blood-brain barrier, its concentration directly reflects the relative blood volume. Thus, activation-induced increases in CBV appear as increased SPION concentration.

Typical SPION doses used in animal fMRI studies are 8–10 mg Fe/kg in macaques [7], and about 5 mg Fe/kg in humans (400 mg of Fe in an 80 kg person), with a maximum of 7 mg Fe/kg or 510 mg total dose [8, 9]. The average human body contains about 5 L of blood.

Given that each cortical brain voxel is 5% blood, a 3 mm isotropic cortical brain voxel thus has 1.35 *μ*l of blood. For a human dose of 400 mg Fe, this corresponds to about 108 ng of Fe in the voxel. Since the CBV increases about 20% during activation [6], we need to be sensitive to about 22 ng Fe. The MPI literature contains sensitivity estimates for rodent scanners as sensitive as 1 pg Fe with high frequency detection, which corresponds to about 50 pg at a conventional (75 kHz) detection frequency [10] although actual animal imagers have not achieved this sensitivity [11, 12]. Thus, if the human imager could achieve the same sensitivity expected for today’s animal MPI, functional activation could be observed with a contrast-to-noise ratio (CNR) of approximately 440, which is quite high compared to fMRI (CNR ~5).

While these estimates are encouraging, they do not account for sensitivity losses incurred in scaling the technology to human head sizes or in reductions from the safety factors needed for human scanners. We show below that the human scanner will have CNR ~40 for a single channel human head-sized solenoid detector, and that a comparable rodent scanner has CNR ~800 with a single channel rodent head-sized solenoid detector. Thus, the penalty for scaling from rodent size to human brain size is about 20×. This factor can roughly be attributed to a reduction in relative detection sensitivity (about 10×) due to the larger receive coils, and a 2× sensitivity drop resulting from using lower drive amplitudes (to avoid peripheral nerve stimulation (PNS)).

## II. Encoding Scheme Studied

The field of MPI instrumentation currently encompasses multiple encoding strategies. Widely speaking, these are based on either Field Free Points (FFP) or Field Free Lines (FFL). Each has variants depending on whether one or more drive fields fulfill the entire role of shifting the FFP/FFL, or if additional, more slowly varying shift fields are used. In either case, multiple reconstruction approaches also exist [13–16].

**Figure 1:**
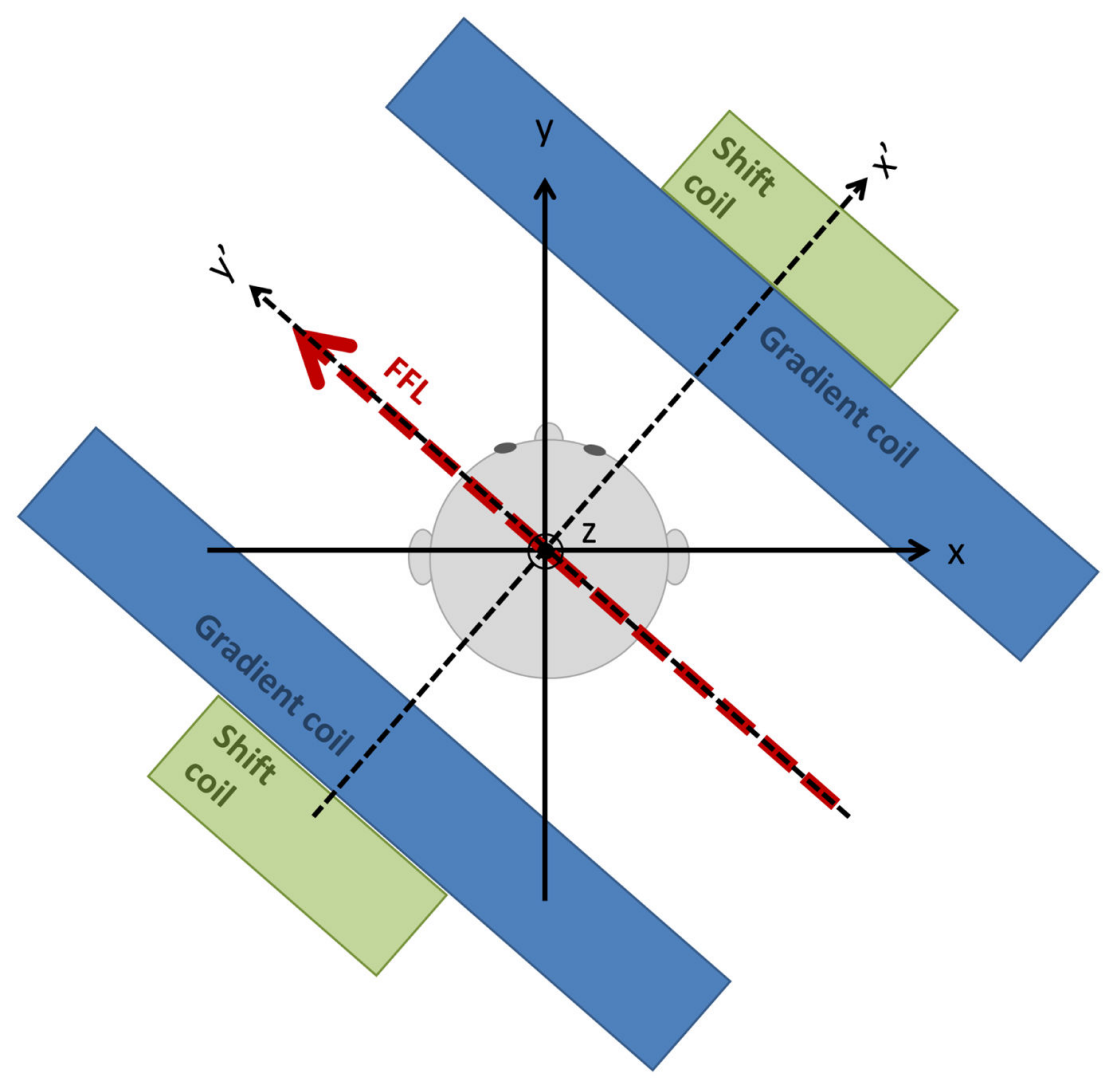
Definition of the coordinate system appropriate for 2D projection-based MPI. The (*x*, *y*, *z*) coordinates are patient centric. The FFL is always along *y*′, which is rotated with respect to the patient. The shift fields always shift the FFL along *x*′ (also rotated with respect to the patient). The drive field is applied along *z*.

In this work, we limit our study to an FFL-based 2D projection scanner, due to its expected sensitivity benefit [17]. A 3D Computed Tomography approach could significantly reduce imaging time [18]; here we use a 2D projection system for the simplicity of only having to shift the FFL in one direction (at the expense of having to rotate the apparatus around the head). The challenge of rotating the apparatus around the subject, however, could employ well-polished commercial gantry and slipring technology developed for CT. The rough geometry of such a scanner is shown in Fig. 1, which omits the drive coil and receive coil. In this configuration, the FFL is shifted along the projection axis, *x*′, acquiring a 1D projection of the iron concentration along this axis. Rotating *x*′ around the patient generates the additional radial projections from which the 2D image is reconstructed. Thus, the coordinate system of the gantry/projections uses the primed axes (*x*′, *y*′, *z*′), and the stationary patient coordinate system is (*x*, *y*, *z*). In this simple CT-like geometry, *z*′ = *z*. Like a spiral CT, additional axial slices can be acquired by translating the patient in the *z* direction.

To preserve design flexibility, we keep the gradient, shift and drive fields separate, although we consider the benefits of combining the gradient and shift fields in the same coil. We keep the drive field separate from the shift fields knowing that PNS will limit the human scanner to far smaller drive fields than those needed to shift the FFL across the head. With a separate coil, the shift fields can slew much slower (1–10 Hz range), avoiding PNS.

For the full-system MPI simulation, we chose to shift the FFL in discrete steps, digitizing multiple cycles of the SPION response at each FFL location in *x*′ followed by rotation to a different projection angle. This simulation is implemented in MATLAB (Mathworks, Natick, MA) with a forward model of the Langevin magnetization and Biot-Savart magnetic field calculations of the coil windings for different gradient, shift, and receive coil geometries. For each point in the projection measurement, we assume that the FFL position is fixed for the duration the signal is recorded. As such, there is no FFL velocity requiring compensation. Each point along the projection axis is recorded by shifting the FFL in discrete steps to its new position. Although likely not the most experimentally expedient approach, we expect similar sensitivities and point-spread functions compared to a continuous approach requiring gridding and velocity compensation. We also assume the shift field can move the FFL across the entire FOV and that multiple cycles of the drive field occur for each FFL position. The latter allows us to record the complex voltage waveform induced in the receive coil (phase and amplitude). Finally, we place the drive field in the *z* direction so it does not interfere with the 2D imaging.

The gradient (or selection) field is the spatially varying magnetic field ***H****_sel_*(*x*′, *y*′, *z*′) that produces an FFL along the *y*′ axis with gradients of strength ***G****_x_* and ***G****_z_* along the *x*′ and *z*′ directions, respectively, such that the gradient field can be expressed as a tensor:

(1) $$H_{\mathrm{sel}}(x^{\prime },y^{\prime },z^{\prime })=G\;\left[\begin{array}{c} x^{\prime }\\ y^{\prime }\\ z^{\prime } \end{array}\right]=\left[\begin{array}{ccc} G_{x} & 0 & 0\\ 0 & 0 & 0\\ 0 & 0 & G_{z} \end{array}\right]\;\left[\begin{array}{c} x^{\prime }\\ y^{\prime }\\ z^{\prime } \end{array}\right].$$

The gradient field is mechanically rotated by gantry rotation, which is represented by the rotation matrix:

(2) $$R(\theta )=\left[\begin{array}{ccc} \cos(\theta ) & \sin(\theta ) & 0\\ -\sin(\theta ) & \cos(\theta ) & 0\\ 0 & 0 & 1 \end{array}\right]$$

such that (*x*, *y*, *z*) = ***R***(*θ*)(*x*′, *y*′, *z*′). Therefore, the selection field in the patient’s frame is:

(3) $$H_{\mathrm{sel}}^{\theta }(x,y,z)=R(\theta )H_{\mathrm{sel}}(x^{\prime },y^{\prime },z^{\prime }).$$

The shift field, $H_{s}^{\prime }(x^{\prime },y^{\prime },z^{\prime })$, is spatially homogeneous and is increased in discrete steps denoted by the index *i* to shift the position of the FFL discretely along the projection axis, *x*′. It also rotates with the gantry so only one coil is needed. Like the selection field, it is rotated into the patient frame using the rotation matrix:

(4) $$H_{s}^{i,\theta }(x,y,z)=R(\theta )H_{s}^{i}(x^{\prime },y^{\prime },z^{\prime }).$$

The simplest depiction of the drive field is a spatially homogeneous, time-varying sinusoid with drive frequency *f*_0_:

(5) $$H_{d}(x,y,z,t)=H_{d}\sin(2\pi f_{0}t)\hat{z}.$$

The receive coil sensitivity profile, ***B***_1_(*x*, *y*, *z*), is defined as the field the receive coil would produce with a unit current applied. By reciprocity, this is related to the receive sensitivity for Faraday detection. These two fields are defined in the patient coordinate system since these coils do not rotate.

In the simulation, we define drive, receive, shift and gradient fields by inputting coil geometries and numerically computing Biot-Savart integrals over each current path. The total field experienced by a SPION is the vector sum of these fields at every vector location and for each point in time. The fields change for each rotation angle *θ* and each discrete shift *i* of the FFL by the shift field along the projection axis.

(6) $$H_{\mathrm{Tot}}^{\theta ,i}(x,y,z,t)=H_{d}(x,y,z,t)+\ldots H_{\mathrm{sel}}^{\theta }(x,y,z)+H_{s}^{i,\theta }(x,y,z)$$

This total field is the input to the Langevin model of the SPION specific magnetization:

(7) $$M^{\theta ,i}(x,y,z,t)=c(x,y,z,t)m\ldots L\;[\beta \left\|H_{\mathrm{Tot}}^{\theta ,i}(x,y,z,t)\right\|]\frac{H_{\mathrm{Tot}}^{\theta ,i}}{\left\|H_{\mathrm{Tot}}^{\theta ,i}\right\|}$$

where *c*(*x*, *y*, *z*) is the concentration distribution of SPIONs, *m* is the magnetic moment, and 1/*β* is the saturation magnetization. The parameters *m* and *β* are best fits to the experimental ***M*(*H*)** curves specific to the SPION sample, as this curve varies with particle diameter, homogeneity, and coating. We used a fit to the experimental data provided by PrecisionMRX^®^ (Imagion Biosystems, Inc., Albuquerque, NM) for their 25nm diameter iron core particles, to determine *m* and *β* in the standard Langevin function.

The voltage induced across the receive coil is the spatial integral of the temporal derivative of this magnetization projected onto the receive coil sensitivity vector (by reciprocity related to the *B*_1_ per unit current generated by the receive coil at that location):

(8) $$v^{\theta ,i}(t)=\mu_{0}\int B_{1}(x,y,z)\cdot \ldots \frac{\partial }{\partial \;t}M^{\theta ,i}(x,y,z,t)dx\;dy\;dz.$$

Although it is not modeled in our simulation, a tuned circuit or impedance transformer in the receive chain would apply a frequency-dependent scaling factor to the voltage. For example, for a human head-sized receive solenoid (i.e. coil (c) in Tab. 1) tuned to the third harmonic, Q is ~400, providing a 400-fold voltage increase of the signal and coil noise.

Noise is approximated by the addition of white Gaussian noise to this digitized signal, *v^θ^*^,^*^i^*(*t*). Noise comes from a combination of body losses, AC losses in the coil conductors, and the preamplifier. We discuss below that the coil losses are likely to dominate for human MPI scanners.

We digitize our signal 200 ksps to acquire frequencies up to 100 kHz, which includes up to the 9th harmonic of the 10 kHz drive frequency. White Gaussian noise representative of the dominant noise source in the system is added to the signal at a level determined by this 100 kHz digitization bandwidth. The desired harmonics are then detected after applying a digital comb filter with a narrower bandwidth, thereby improving the detection sensitivity. This filtering is modeled by selection of specific frequency components of this signal via a Fourier transform and a frequency component selection operator, ***Ô****_s_*. For instance, selecting only the 3rd harmonic frequency would be represented as:

(9) $$v_{3}^{\theta ,i}(t)={\hat{O}}_{3}\left\{F\;\left\{v^{\theta ,i}(t)\right\}\right\}.$$

The sum of the selected frequency components, $v_{s}^{\theta ,i}(t)$, is assigned to point *i* of the projection, and this is done for each shift of the FFL along the projection axis. This forms a single projection, and the process is repeated for each rotation angle *θ*.

**Figure 2:**
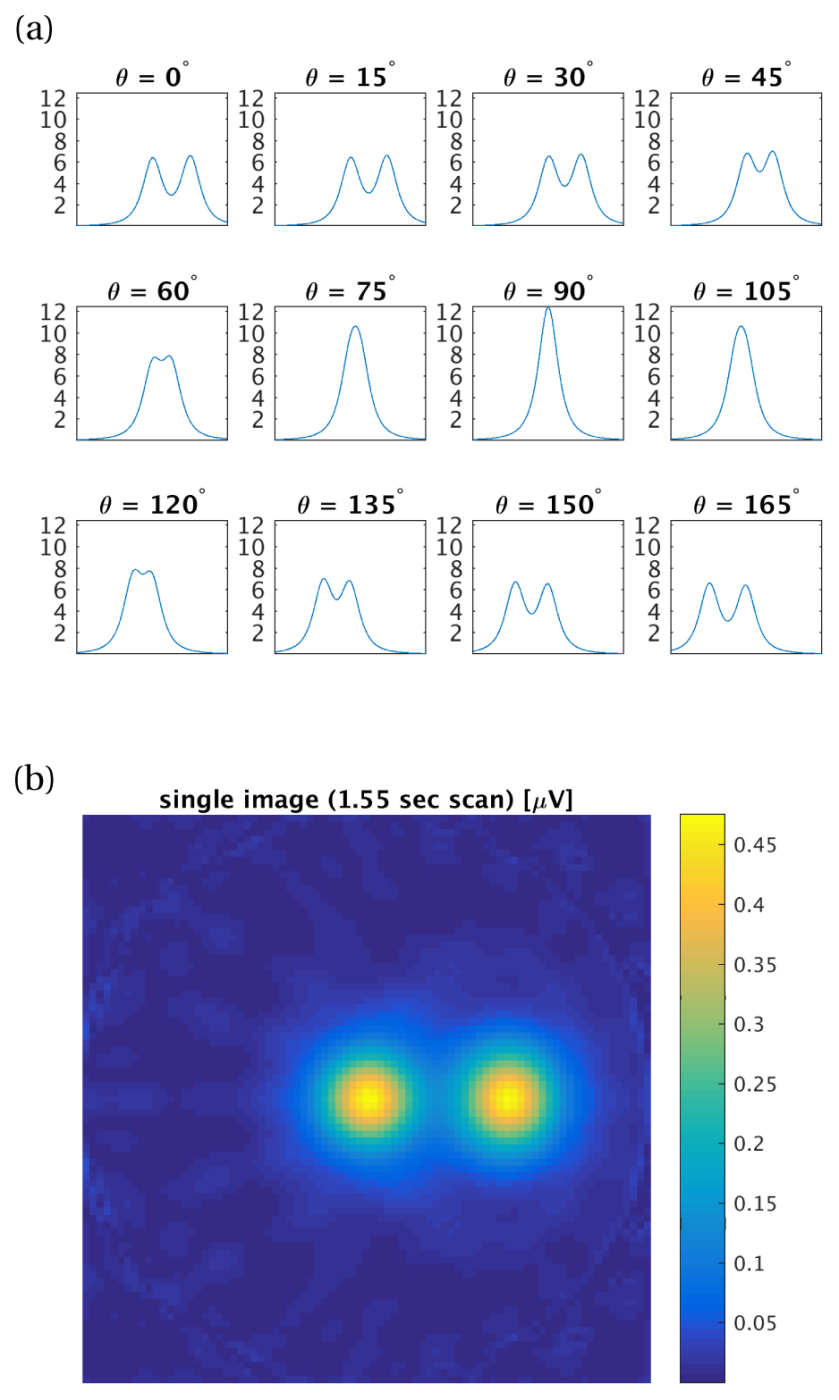
(a) Simulated projections [*μ*V] for 12 angles from 0° to 180° about two 50 *μ*g Fe samples in 2.3 mm^3^ volumes located at (*x*, *y*, *z*) = (0,0,0) and (5 cm,0,0). Drive field: 25 mT at 10 kHz, gradient FFL: 1.5 T/m. Projection axis: [−10 cm, 10 cm], 81 points. Signal received with human-head size solenoid receive coil with 25 turns (coil (c) in Tab. 1), signal filtered to 3rd harmonic. Added noise corresponds to Nyquist noise in receive coil at sampling BW100 kHz (9.77 nV). 1.6ms scan per point, 1.55 sec imaging time. (b) Reconstructed axial slice image using the 12 projections from (a).

**Figure 3:**
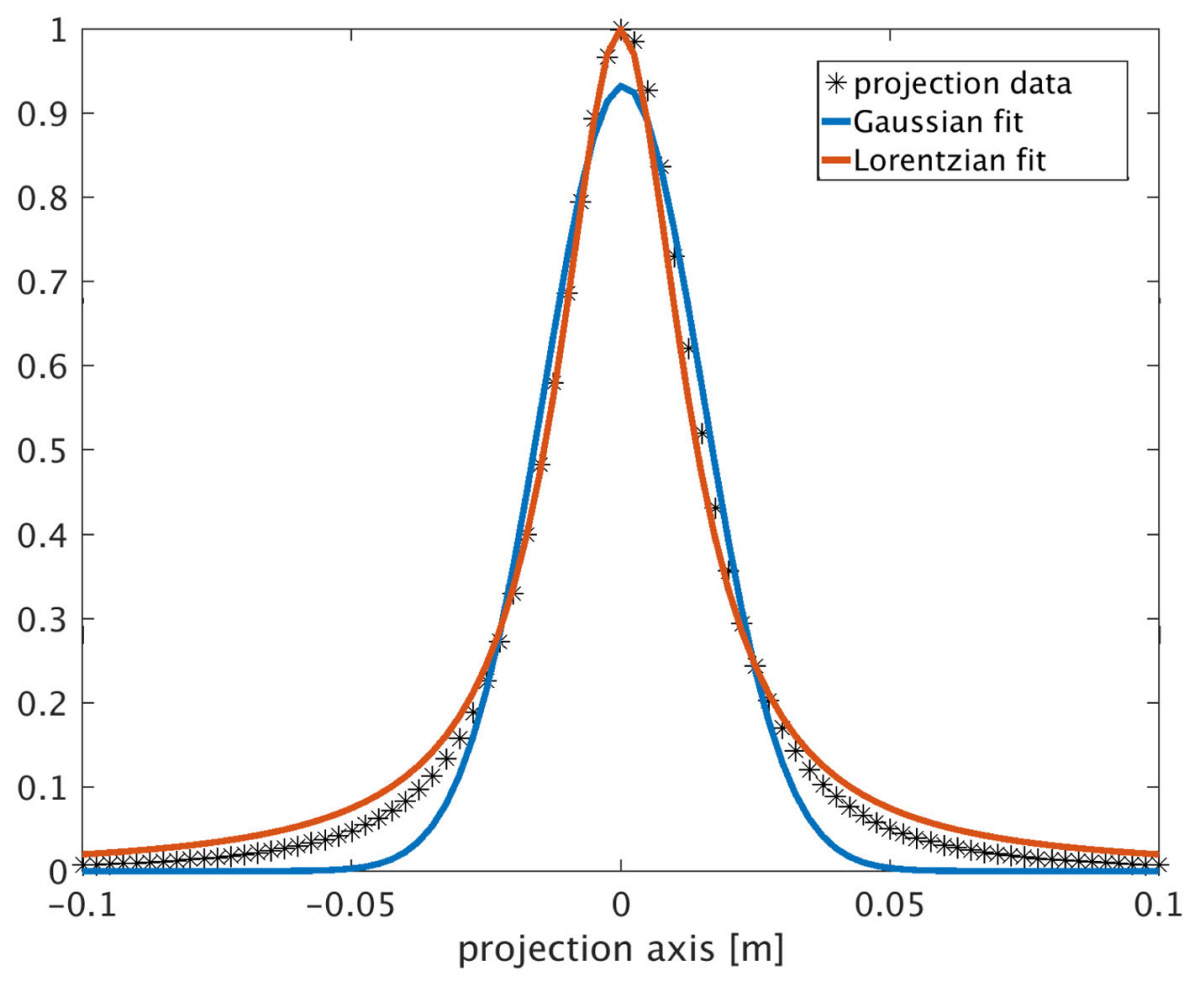
Single projection (1 angle) of 22 ng Fe sample at center in 2.3 mm^3^ volume; 81 points in projection axis; each point in the projection is the 3rd harmonic frequency component of a 1.6ms scan of a 25 mT 10 kHz drive field. Gradient strength is 1.5 T/m. No noise added. Receive coil: human-head size solenoid, 25 turns (coil (c) in Tab. 1). Gaussian and Lorentzian distributions are fitted to this projection (normalized).

Fig. 2(a) shows projections at 12 angles formed with a 25 mT drive field at 10 kHz, of two 50 *μ*g SPION samples. The Langevin curve is fit to magnetization data for 25nm PrecisionMRX^®^ particles (Imagion Biosystems, Inc., Albuquerque, NM), per kg elemental Fe. The receive coil is a head-sized uniform solenoid, the FFL has gradient strength |*G_x_*| = |*G_z_*| = 1.5 T/m. The shift field scans the FFL ±10 cm. The 12 projections from Fig. 2(a) are reconstructed to form the axial slice image shown in Fig. 2(b). The reconstruction can be done with methods such as filtered back-projection; here, they are reconstructed by minimizing the least-squares data consistency error to the forward projection model. A projection from a single 22 ng Fe sample at (*x*, *y*, *z*) = (0, 0, 0) is shown in Fig. 3, fitted to both Gaussian and Lorentzian functions. The projection axis is discretized into 81 points and the signal is normalized to the maximum signal in the projection.

## III. Shift/Gradient Field Hardware

The gradient strength of the field-free line magnet, together with the SPION’s Langevin transition width, governs the achievable spatial resolution. The FFL can be produced by either i) combinations of permanent magnets, resistive electromagnets, and iron, or ii) superconducting electromagnets.

**Figure 4:**
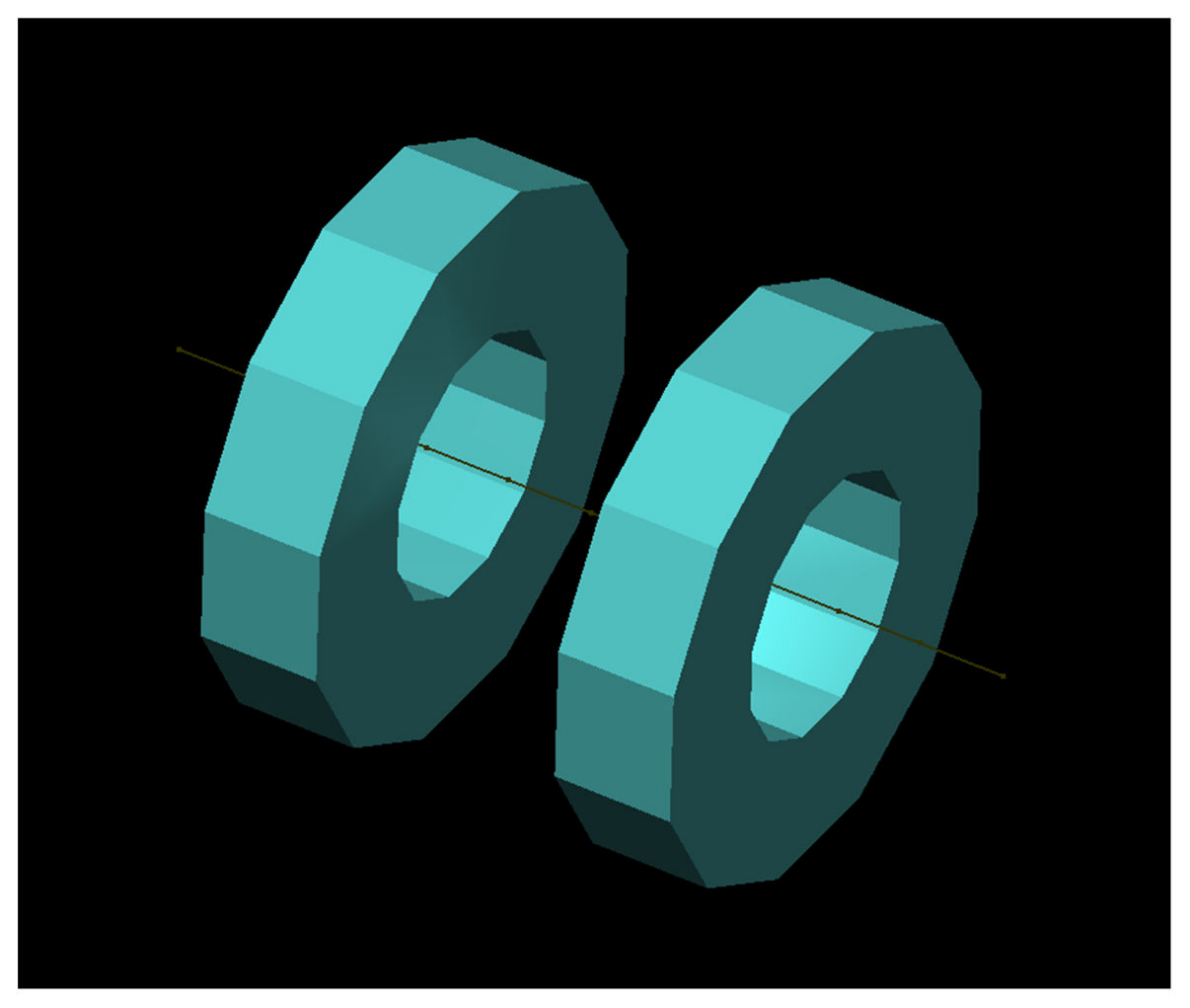
Maxwell pair of electromagnet coils from GMW Associates. Coil diameter *d* = 30 cm, spacing = *d*/2. Driving each 360-turn coil with 140 A produces 0.13 T max field, forming a 1.7 T/m gradient along the axis.

**Figure 5:**
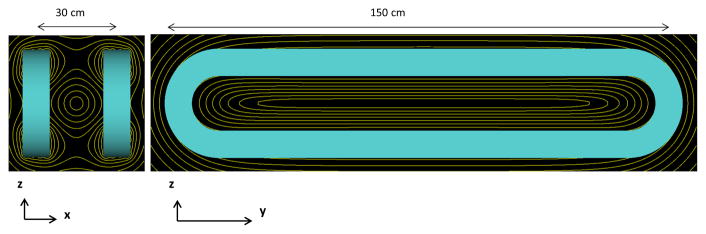
Electromagnetic gradient coil configuration of two racetrack coils, 360 turns each, long axis = 150 cm, short axis = 30 cm, separated by 30 cm. 140 A applied. Simulated B field isolines shown in yellow. *G_x_* = *G_z_* = 0.7 T/m.

Commercially available resistive electromagnet coils provide insight to the achievable gradient strength, power supply and cooling needs easily available from commercial coils. GMW Associates (P/N11801653, GMW Associates, San Carlos, CA) produces a 0.13 T (at 140 A) electromagnet coil with inner diameter 30.6 cm [19]. Two of these coils arranged in a Maxwell pair configuration (Fig. 4) would produce a 1.7 T/m gradient along their mutual axis. These circular coils do not produce a field free line, which would require a more eccentric winding pattern, as described below and shown in Fig. 5. Additionally, the ~15 cm spacing is insufficient for the human head. Nonetheless, this shows that a gradient strength around 1–2 T/m is readily attainable at a near human scale. Each of these coils dissipates 5.18W at peak operation, and uses water-cooling with the flow of 15 liters/min at 1.0 bar pressure.

A full electromagnet human-sized gradient with an eccentric winding pattern to create an FFL is shown in Fig. 5. It comprises two oval coils arranged in a Maxwell-like configuration. The long axis of each coil is 150 cm in length and the short axis is 30 cm, with a 30 cm spacing between the coils. Based on the GMW electromagnet windings and cooling specifications, we consider each coil with 360 turns and a current of 140 A applied to each. The gradient strength achieved along both directions transverse to the field-free line is 0.7 T/m. One benefit of an electromagnetic gradient coil is the option to integrate shift fields needed to move the position of the FFL by modulating the current to each of the two coils. An electromagnetic gradient coil could also include an iron yoke to focus the field.

Superconducting wire with cryogenic cooling is another design possibility, allowing the design flexibility to increase the current to achieve higher gradient strength. AC superconductor performance (needed if the same windings will also be used to dynamically shift the FFL) is relatively limited compared to DC operation, but conductors with good current performance up to 20 Hz are available. An AC superconductor (Nb_3_Sn) paired with Litz wire to better transfer heat has been proposed as a “smart bobbin” for AC magnets and has been shown on a 20 cm cylinder [20]. The technology allows 100 A at 20 Hz and 8 K with sub-mm diameter wire. The challenge with AC superconductors is removing the heat generated from AC losses, which typically amounts to 1–5Wfor such a coil. Available cryocoolers limit this to ~5W. Thus, the expected performance for AC superconductors is roughly analogous to what can be achieved with conventional copper electromagnets. The copper electromagnet generates three orders of magnitude more heat, but this is addressed with cheap and efficient water-cooling. Note that while the gradient generated using a DC superconducting coil could be quite high, it would require AC coils to dynamically shift the FFL (requiring resistive or AC superconductive windings).

**Figure 6:**
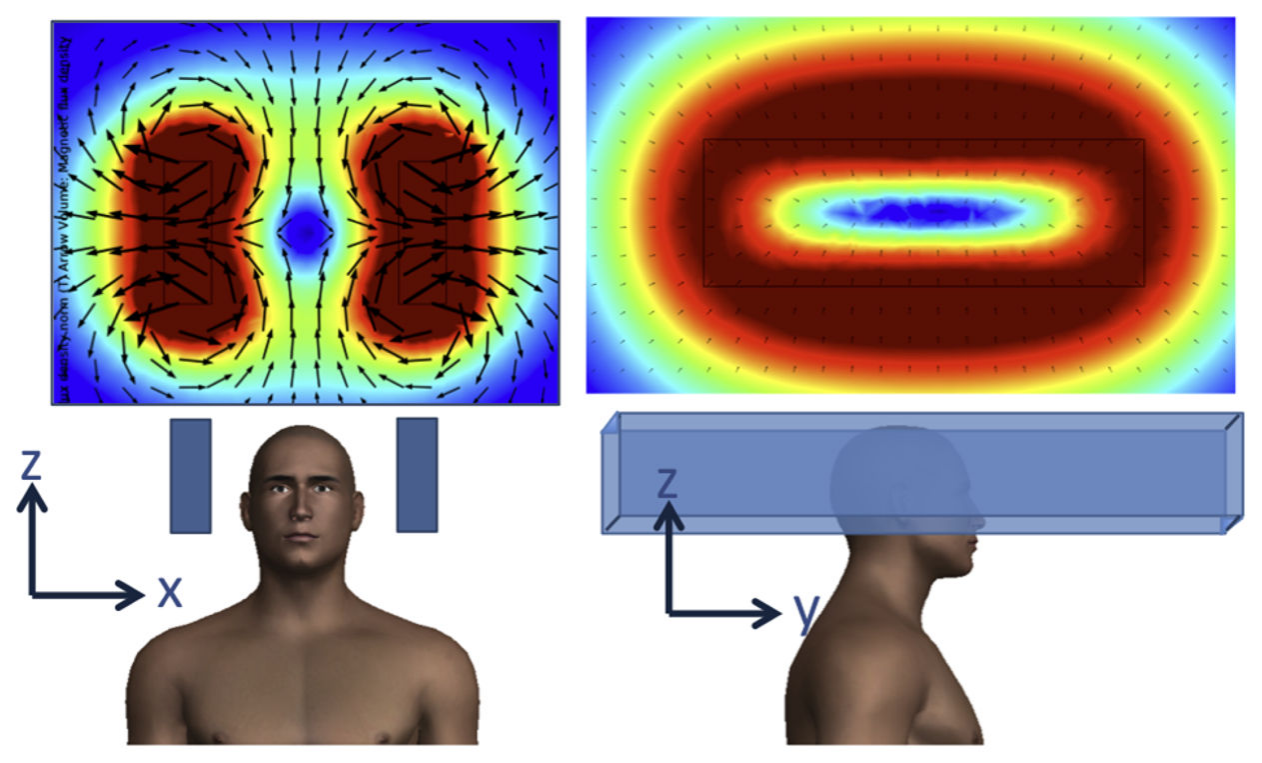
Permanent magnet-based FFL designed to rotate about the head (about *z*-axis). 2″×4″×36″ N48 magnets produce FFL with *G_x_* =*G_z_* = 1.0 T/m.

**Figure 7:**
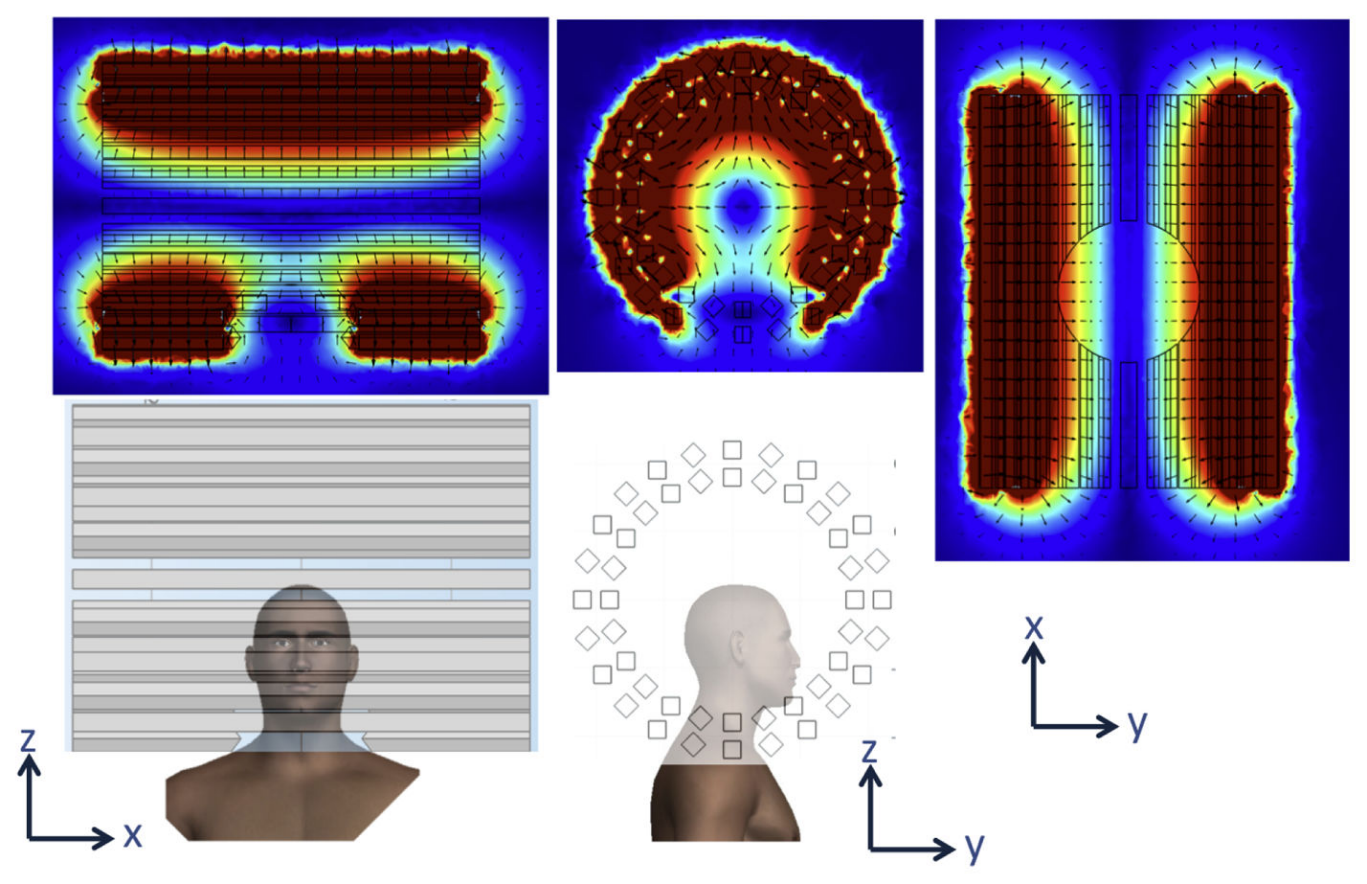
Permanent magnet-based FFL designed to rotate about the head (about *z*-axis). *K* = 3 Halbach array of N48 magnets with opening for the head produce FFL with *G_x_* =*G_z_* = 1.5 T/m.

**Figure 8:**
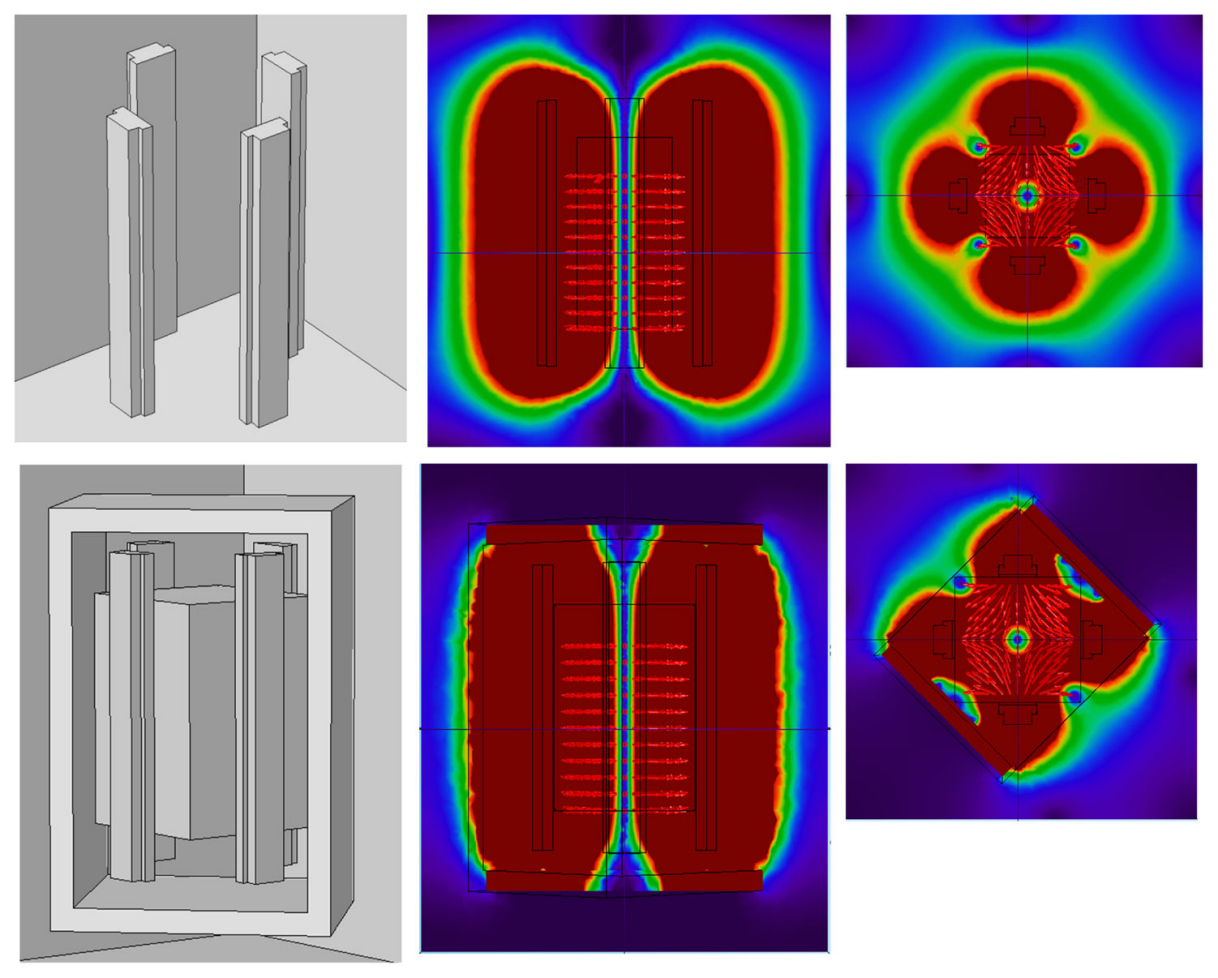
Permanent magnet-based FFL designed to rotate about the head. Quadrupole arrangements of ~4″×2″ N52 magnets produce *G_x_* = *G_z_* = 0.9 T/m without the iron yoke, and *G_x_* =*G_z_* = 1.2 T/m with the iron yoke.

Permanent magnets are attractive because they do not need power nor generate heat, and can create FFL gradients of about 1.5 T/m in head-sized geometries. However, they would require separate shift field electromagnets to move the FFL across the head or some sort of mechanical translation. Preliminary designs utilizing permanent human head-sized magnets have been simulated in COMSOL and are shown in Fig. 6, Fig. 7, and Fig. 8. Fig. 8 includes a magnet with and without an iron yoke. The yoke increases FFL gradient from 0.9 T/m to 1.2 T/m.

In summary, a gradient system with about 1.5 T/m is likely feasible for a head-sized FFL type MPI apparatus. Rodent MPI scanners with a gradient of 7 T/m currently achieve about 1.5 mm spatial resolution, and a 6.3 T/m projection MPI scanner has been proposed with theoretical spatial resolution of 600 *μ*m[12]. The gradient scaling from 7 T/m in rodent to 1.5 T/m in humans suggests a human scanner will have approximately a 5-fold reduction in spatial resolution compared to current rodent MPI scanners, suggesting an achievable spatial resolution of 7 mm, which is comparable to the resolution of spatially-smoothed fMRI images.

Shifting the FFL by ±10 cm in a 1.5 T/m gradient will require a shift field of ±150 mT. This is within the achievable range for electromagnets as well as AC superconducting coils. Tolerance by the patient is another concern. Oscillating a field of this magnitude at even a few Hertz (to generate rapid projections) is likely to cause retinal stimulation (magneto-phosphenes). Magnetostimulation of the retina has well-studied thresholds, and the human eye is most sensitive at frequencies of ~20 Hz, where the threshold for phosphene generation is about 40 mT [21]. Expressed as a dB/dt, this 20 Hz field has a maximum dB/dt of 5 T/s. While generating a few phosphenes is not considered a safety concern, the retina is a metabolically delicate organ, and sustained excitation may lead to excitotoxic damage, although very few studies have been performed to investigate this concern. For a human fMPI system using a 1.5 T/m gradient in which 53 projections are acquired to form an image in 3 seconds, the shift field has a dB/dt of 5.4 T/s, near the magnetostimulation threshold. Increasing the duration of the FFL sweep across the head to about 1 second would decrease this dB/dt about 20-fold, but would also slow down the imaging to ~1 second per projection, which is too slow for functional studies. Therefore, our temporal resolution is limited to about 3 seconds per image in order to adhere to this magnetostimulation threshold (given 53 projections are required per image, the FFL strength is 1.5 T/m, and the FFL is shifted across a 20 cm FOV). Since hemodynamic changes to brain activation take place on the 3–6 sec timeframe, this temporal resolution should be sufficient for functional MPI and is comparable to that used by the majority of fMRI studies.

An alternative approach is to utilize partial projections whereby the FFL is not fully swept across the head, but perhaps only as little as ±2.5 cm across the central region of the head. This would reduce the shift field amplitude by a factor of four, but constitute an image reconstruction challenge. The use of “interior reconstructions” to reconstruct the central part of the FOV that is covered by the partial projection has a long literature [22, 23]. Thus, one approach is to place this region on the area of activation and only image part of the brain. A second approach is to try the more difficult reconstruction of not just the interior region but the whole head using only truncated projections and parallel reception information. We have introduced preliminary work that this might be feasible [24].

In addition to dB/dt imposed by the shift field, rotation of the gantry containing the FFL also causes dB/dt in the retina. If the gantry containing a 1.5 T/m gradient FFL were rotated at 1 Hz, the eyes, 10 cm from isocenter, would experience dB/dt = 1.2 T/s. Thus, we do not expect the gantry rotation to cause retinal stimulation (it is well below threshold for the worst-case frequency).

## IV. Drive Field Requirements

**Figure 9:**
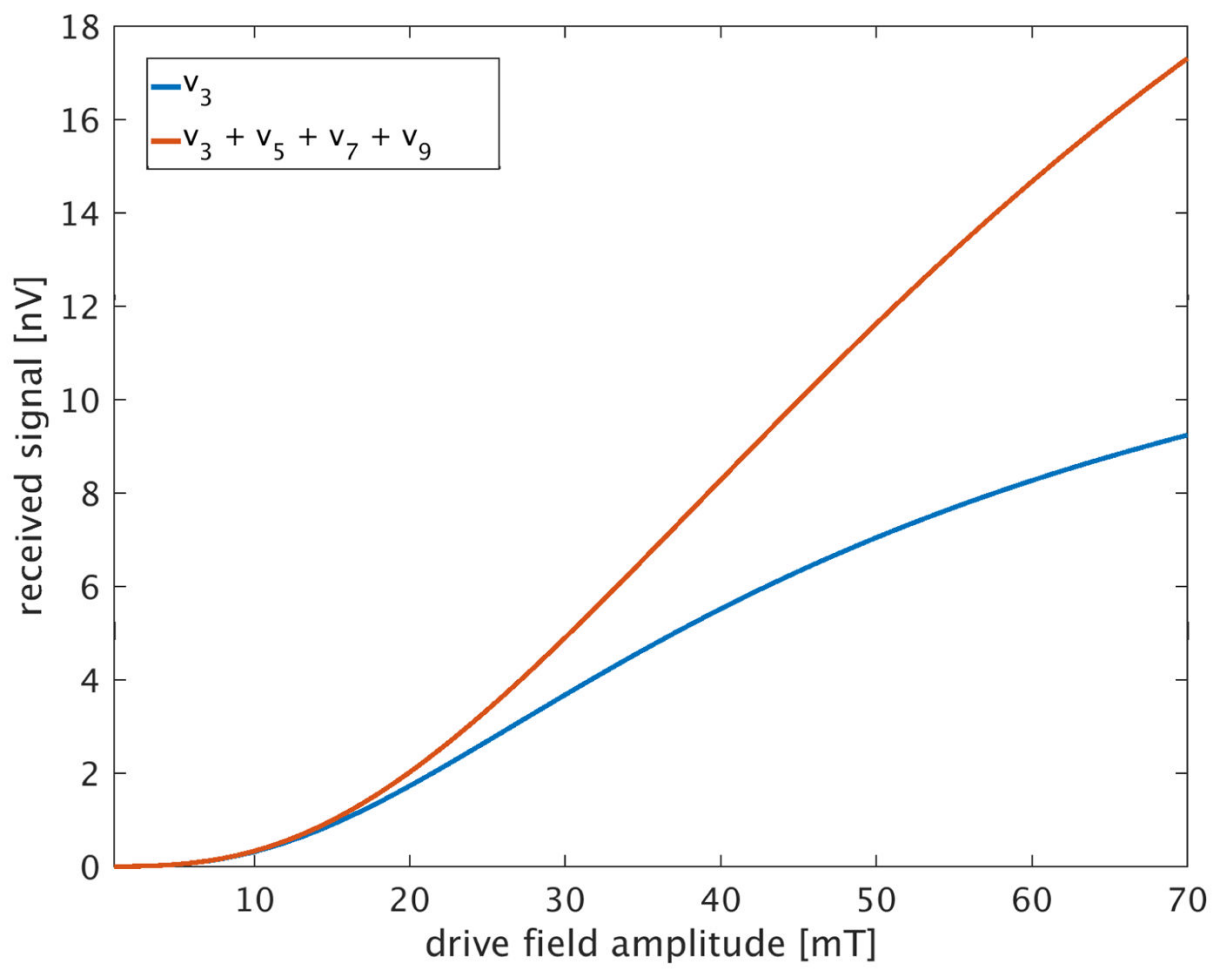
Comparison of frequency component contributions to received signal versus the 10 kHz drive field amplitude. Input object is 22 ng Fe sample at center in 2.3 mm^3^ volume. A 1.6ms scan measures the signal at the center point in projection axis. No noise added to signal. Receive coil is human head-sized solenoid (*r* = 12 cm, *l* = 24 cm, 25 turns; coil (c) of Tab. 1). Blue curve is the 3rd harmonic frequency component; orange curve is the sum of the 3rd, 5th, 7th and 9th harmonic frequency components, for drive field amplitudes from 1 mT to 70 mT. With a tuned circuit or impedance transformer in the receive path, the voltages received will be “stepped-up” to a considerably higher value at the preamplifier input.

To induce sufficient nonlinear signal from SPIONs, the particles must be driven into their magnetic saturation regime. Fig. 9 shows the simulated received signal (3rd harmonic component only, and sum of 3rd–9th odd harmonic components) as the drive field amplitude is increased from from *H_d_* = 1 to 70 mT for the PrecisionMRX^®^ 25 nm particles. This relationship is monotonically increasing and nonlinear, and differs depending on the frequency harmonics detected.

While increasing the drive field amplitude will increase the nonlinear response of the SPIONs and thus the sensitivity, an upper bound on practical drive strength is set by PNS safety limits in humans. Human MPI drive fields will likely operate in the tens of kHz, in the regime where PNS effects dominate over specific absorption rate (SAR) effects [25]. Work by Saritas et al. [25] places these limits for the torso at 9.9 mT at 4.5 kHz and 7.6 mT at 25 kHz. From fast imaging in MRI, we know that head-only coils will likely have limits 2–3× above torso limits. Nonetheless, the PNS will set a bound on safe drive field strengths and adversely affect the sensitivity. We anticipate the sensitivity of a human head MPI will be reduced ~2-fold compared to rodent scanners due to this effect.

Thus, there is a tradeoff between drive frequency, PNS limits and detection sensitivity (since Faraday detection efficiency is proportional to the frequency). In general, PNS thresholds increase at lower drive frequencies. Bozkurt and colleagues [26] have shown that, for the PNS limited case (likely for human work), the detection sensitivity is rendered nearly independent of drive frequency due to this tradeoff. In short, lower drive frequency provides less sensitive Faraday detection, but allows a higher drive field amplitude, which makes up for this loss.

The excitation/drive field is ideally a spatially homogeneous sinusoid with an amplitude sufficient for SPION saturation but low enough to adhere to safety limits and maintain patient comfort. A spatially homogeneous field on the order of tens of mT is producible with a solenoid drive coil (concentrically exterior to the receive coil/array) tuned to the drive frequency, and amplified with a standard commercially available gradient amplifier.

The choice of SPION, with its specific Langevin curve, will impact the drive field/sensitivity tradeoff, and will also determine achievable spatial resolution. Development of improved SPIONs will be an important part of getting the maximum sensitivity and spatial resolution out of the human MPI scanner. SPION development is an active area of research that is outside the scope of this design analysis [27, 28].

## V. Receive Strategies

Signal acquisition in MPI utilizes Faraday detection of the time-varying SPION magnetization induced by the excitation field. Like MRI, the optimal Faraday detector has enough sensitivity to be limited by random ionic currents in the body (body-load noise) rather than Johnson-Nyquist noise from losses in the coil, or from the added noise from the preamplifier. While the receive goal of MPI is sample-noise dominance, this is difficult at the low detection frequency of human MPI (<100 kHz) where sample noise is small compared to the Johnson-Nyquist noise from losses in the coil components (“coil noise”) or noise added by the preamp (“preamp noise”). The crossover frequency for body noise dominance for Faraday detection with room temperature coils is about 25 MHz [29]. Even with high *T_c_* superconductive cooled coils, this crossover occurs at about 4–10 MHz for a 65 cm loop [29]. Therefore, body noise is not likely to be the dominant source of noise for room temperature MPI coils receiving at frequencies less than 100 kHz. Low frequency MRI coils have benefitted from cooled copper or superconducting circuits [30]. This strategy is likely also beneficial for human MPI.

Traditional Faraday detectors can be either tuned or untuned. One option for MPI is to tune the receive coil to a single frequency, such as the 3rd harmonic. This system would be sensitive to this strongest component of the nonlinear SPION signal (the drive frequency component of the particle’s magnetization is masked under the drive field’s induced voltage, rendering it difficult to detect). Although the higher harmonics contain signal power, focusing on a single harmonic would allow a high Q coil, although coils could be simultaneously tuned to multiple resonance frequencies.

We note that standard commercial low noise preamplifiers such as the SR560 (Stanford Research Systems, Sunnyvale, CA) have a noise voltage referred to the input of about $4\;\text{nV}/\sqrt{\text{Hz}}$, which is 1.26 *μ*V for a 100 kHz BW. Recently published room-temperature MPI preamplifiers have demonstrated a noise level of about $1\;\text{nV}/\sqrt{\text{Hz}}$ (about 316 nV in a 100 kHz BW) [31]. Commercial cryogenic preamplifiers (NexGen3, Stahl-Electronics, Mettenheim, Germany) have even better noise performance, about $0.4\;\text{nV}/\sqrt{\text{Hz}}$ (126 nV in 100 kHz BW). We consider only preamp voltage noise here and do not analyze current noise independently, based on the assumption that noise matching will be achieved, balancing the two.

The receive coil typically utilizes a step-up transformer to match the sub-Ohm coil resistance to the desired load that optimizes preamp noise performance. Typically, this optimum load is many kΩ. The transformer steps up both the signal and the coil/body noise voltage by more than a factor of 10. This is enough to render the preamplifier noise relatively unimportant for Faraday detection at these frequencies [29]. Thus, the dominant noise source for room temperature receive coils comes from losses in the coil’s conductors. Other sources of noise, such as thermal losses in the body, are also considered but tend to be small compared to the other sources (see Tab. 1).

Tab. 1 considers room temperature receive coil designs, listing estimates for the sample and AC conductor-loss noise voltages as well as the *B*_1_ efficiency (Faraday detection efficiency). We compare solenoids, Helmholtz pairs, and multi-channel array receive designs using Litz wire for both rodent- and human-scale MPI systems.

We note that our noise analysis neglected losses in any filter or impedance transformation device in front of the preamplifier. Any losses prior to the preamplifier add noise to the final measurement and degrade SNR. A notch filter, however, may be necessary to avoid saturating the preamplifier with drive-frequency feedthrough. Similarly, an impedance transformation device to transform the detection circuit to the impedance desired by the preamplifier is essentially implicit in our assumption that the noise is coil-loss dominated. The amount of loss added depends on the filter design specifics. But, as with the receive coils, it is likely that the principal loss source is ohmic losses in the Litz wire of the inductors. Thus the ratio of receive coil losses to filter/impedance transformation losses is given by the relative length of wire used for each. For the human-sized coils studied, the length of wire needed was 18 m (coil (c) of Tab. 1) and 36 m (coil (d) of Tab. 1), so considerable wire lengths could be used in the filter inductors before adding significantly to the losses.

The power lost in the body resistance for a given applied *B*_1_ reciprocity field is estimated by integrating the square of the electric field over the lossy object [32]. For a coil generating a uniform effective *B*_1_ field, and uniform conductivity object, the power dissipated in the body scales as the square of the frequency, the 4th power of the cylinder diameter, and the length of the cylinder [32]:

(10) $$P_{\text{sample}}=\frac{1}{8}\omega^{2}\sigma B_{1}^{2}\int_{\text{sample}}r_{⊥}^{2}dv$$

where $\int_{\text{sample}}r_{⊥}^{2}dv=\frac{\pi }{32}D^{4}l$ for a cylindrical sample of length *l* and diameter *D*, with an RF field oriented parallel to the axis of the cylinder. Thus:

(11) $$P_{\text{sample}}=\frac{1}{8}\omega^{2}\sigma B_{1}^{2}\frac{\pi }{32}D^{4}l.$$

We approximate the human head object as a cylindrical sample of diameter 22 cm, length 22 cm and conductivity 0.5 S/m, and a rodent head as a cylindrical sample of diameter 4.5 cm, length 4.5 cm, and conductivity 0.5 S/m. For this effective resistance, we compute the noise voltage across this resistor in a 100 kHz receive BW using the standard formula for Johnson-Nyquist noise in a resistor (Tab. 1).

We also estimate the AC losses in the coil itself and similarly convert this resistance into a noise voltage. Our calculations assume 4 parallel strands of Litz wire with equivalent gauge 26 AWG (New England Wire, Lisbon, NH, USA) and DC resistance per length *R* = 44Ω/1000 Ft, and calculate the AC resistance using an AC-to-DC conversion factor defined by New England Wire [33] at 30 kHz (third harmonic frequency). We then compute the Johnson-Nyquist noise for this AC resistance in the 100 kHz bandwidth and at a temperature of 273 K.

Together these calculations show AC conductive losses in the coil produce more noise than the body losses for all of the room temperature receive coil geometries examined. But, with cryogenic coil cooling, sample noise dominance could be feasible. Cooling the conductor will be effective at reducing the coil resistance until approximately the Debye temperature, at which point impurity content dictates the conductivity. Thus, this strategy might be effective down to LN_2_ temperatures where a gain of about 300 K/77 K = 3.9 could potentially be achieved. Alternatives to traditional Faraday detection include magnetometer-based detection (of the field rather than the temporal derivative of the flux) using SQUID or optical Faraday rotation magnetometers.

## VI. Sensitivity Simulation

Using the MPI simulator described above, we explore the detection limits of SPION samples for a human head functional imaging system (1.5 T/m gradients). Acquiring an image every ~3 seconds, we mimic a ~10 minute fMPI study by assessing the sensitivity of 200 averages of this single 3 sec image. To form the 2D projection image, the gantry is rotated 180 degrees. A minimum of 53 angles across this 180 degrees and 35 points per projection are required to reconstruct the image with 6 mm resolution over a 20 cm FOV. This allows us to sample the magnetization response for a duration of 1.6ms per point in the projection (16 periods of the 10 kHz drive frequency). The exact scan time is 2.97 sec per timepoint; for 200 averages, this is a total imaging time of 593.6 sec (~10 min).

**Figure 10:**
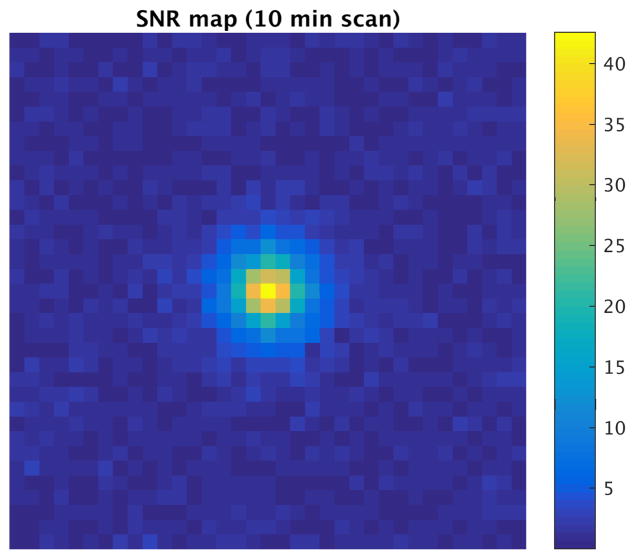
SNR map of 22 ng Fe sample placed in 2.3 mm^3^ region at center of human-head fMPI system. 53 projections and 35 points along 20 cm projection axis give 6 mm resolution. 3 sec scan time per image; 200 averages. Driven by 25 mT sinusoid at 10 kHz. Gradient strength is 1.5 T/m. Receive coil: human head-sized solenoid (coil (c) in Tab. 1).

We simulate projections and reconstruct an SNR map image of a 22 ng Fe sample in a 2.3 mm^3^ region at the isocenter (Fig. 10). This 22 ng Fe sample reflects the expected change of iron in a 3 mm×3 mm×3 mm cubic section of cortex during activation, as discussed previously. We assume the noise for this system is dominated by AC conductive losses in the coil windings, and the signal is received with a 25-turn human head-sized solenoid (coil (c) in Tab. 1); thus, noise added to the simulated received signal is the Nyquist noise in this coil in our 100 kHz digitization bandwidth (9.77 nV white Gaussian noise). The peak SNR is 42, indicating that fMPI has sufficient sensitivity with completely conventional MPI approaches to outperform fMRI (which typically sees activation with about CNR = 5), but bigger sensitivity benefits of fMPI will require improvements in the SPIONs and/or receive methods. If the activation source is distributed (e.g. over a cortical region), then the lower spatial resolution of the human imager translates to a larger weight of iron in the voxel, which will improve its SNR.

We also use this simulation to investigate the SNR loss expected from scaling the system up from rodent to human size. In this comparison, we simulate acquiring projections of the same sample, 22 ng Fe, with both rodent and human systems. The rodent system uses a solenoid receive coil (Tab. 1, (a)) with a 2.5 cm radius, 5 cm length, and 25 turns. The gradient field achievable at the rodent scale is larger; an FFL of 7 T/m is used. The system is driven with a 50 mT sinusoidal field at 10 kHz. 35 points are acquired for each projection covering 4 cm in the rodent system, and 53 projections allow Nyquist sampling of the projection space to achieve ~1.18 mm spatial resolution. The rodent scheme also uses 1.6 ms of digitization with a 200 kHz sampling rate for each point in the projection, and the signal is filtered to select only the 3rd harmonic component. Coil losses for the rodent-sized solenoid are represented by the addition of 4.46 nV white Gaussian noise (Nyquist noise of coil (a) of Tab. 1 at 100 kHz BW).

**Figure 11:**
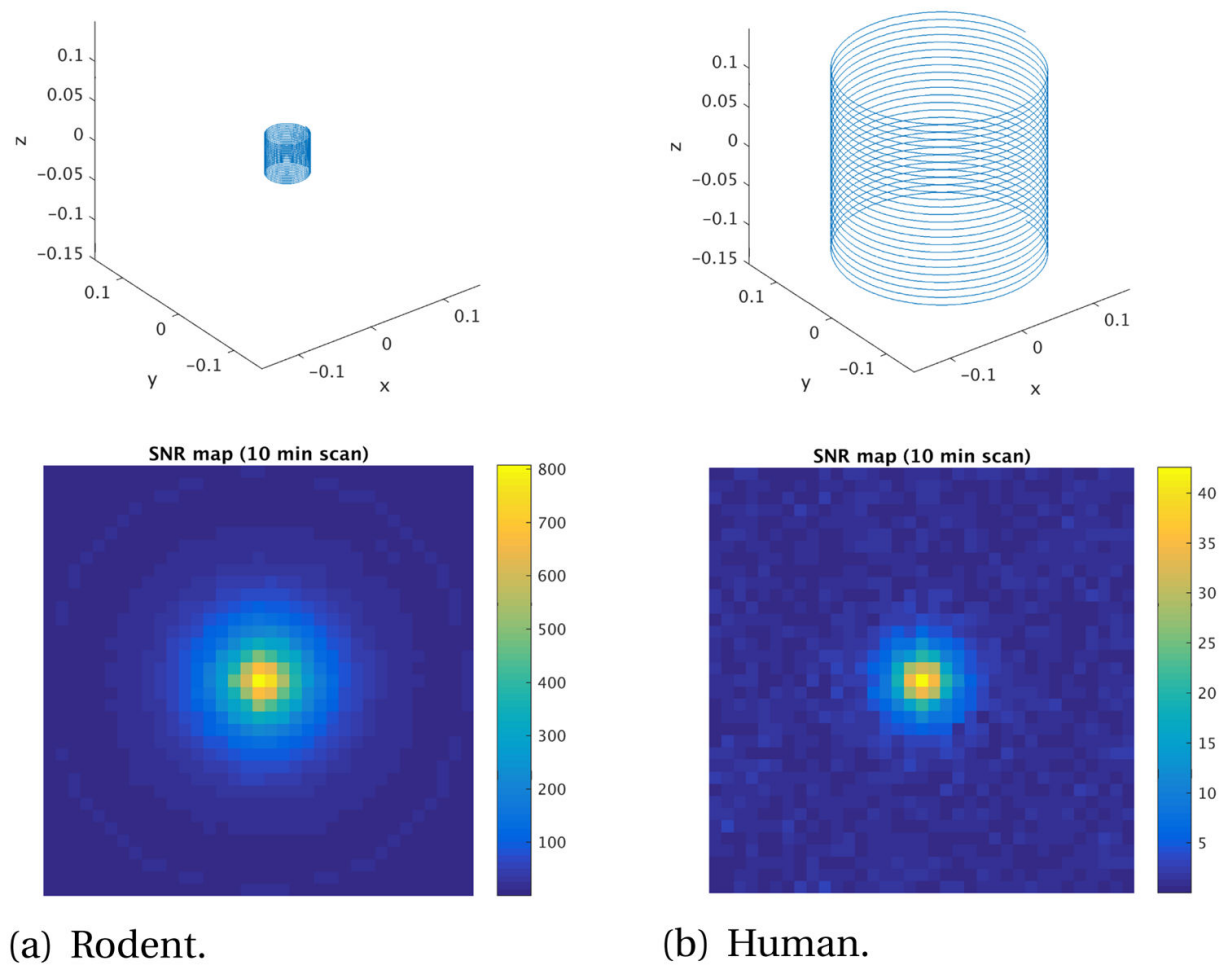
Comparison of rat- and human-sized solenoid receive coils and respective reconstructed images of a 22 ng Fe sample in a 2.3mm^3^ region at the center of the FOV. For both, the axial slice imaged is *z* = 0, 53 projections are acquired at gantry rotations equally spaced between 0° and 180°, and 35 points are taken along the projection axis. (a) Rodent system: receive coil (a) from Tab. 1, *B*_1_ at center = 444 *μ*T/A, AC conducting noise = 4.46 nV. 4 cm FOV. Gradient FFL = 7 T/m×7 T/m. Drive field = 50 mT at 10 kHz. Max SNR = 807.5. (b) Human system: receive coil (c) from Tab. 1, *B*_1_ at center = 93 *μ*T/A, AC conducting noise = 9.77 nV. 20 cm FOV. Gradient FFL = 1.5 T/m×1.5 T/m. Drive field = 25 mT at 10 kHz. Max SNR = 42.6. SNR ratio = 19.

The ratio of relative detection sensitivities between rat and human is ~10 (Tab. 1), and the drive field of the rat is a factor of 2 higher, suggesting an SNR scaling factor of ~20 between rodent and human systems. This agrees with the simulations comparing rodent and human systems for imaging a point source (Fig. 11), where the 22 ng source was seen with SNR = 807 while the human imager detected with SNR = 42, a ratio of about 20. The simulated rodent sensitivity also roughly agrees with that expected for animal scanners (~50 pg); note that the simulations suggest a 30 pg sample (22 ng/807) is detectible in 10 min with SNR = 1.

**Figure 12:**
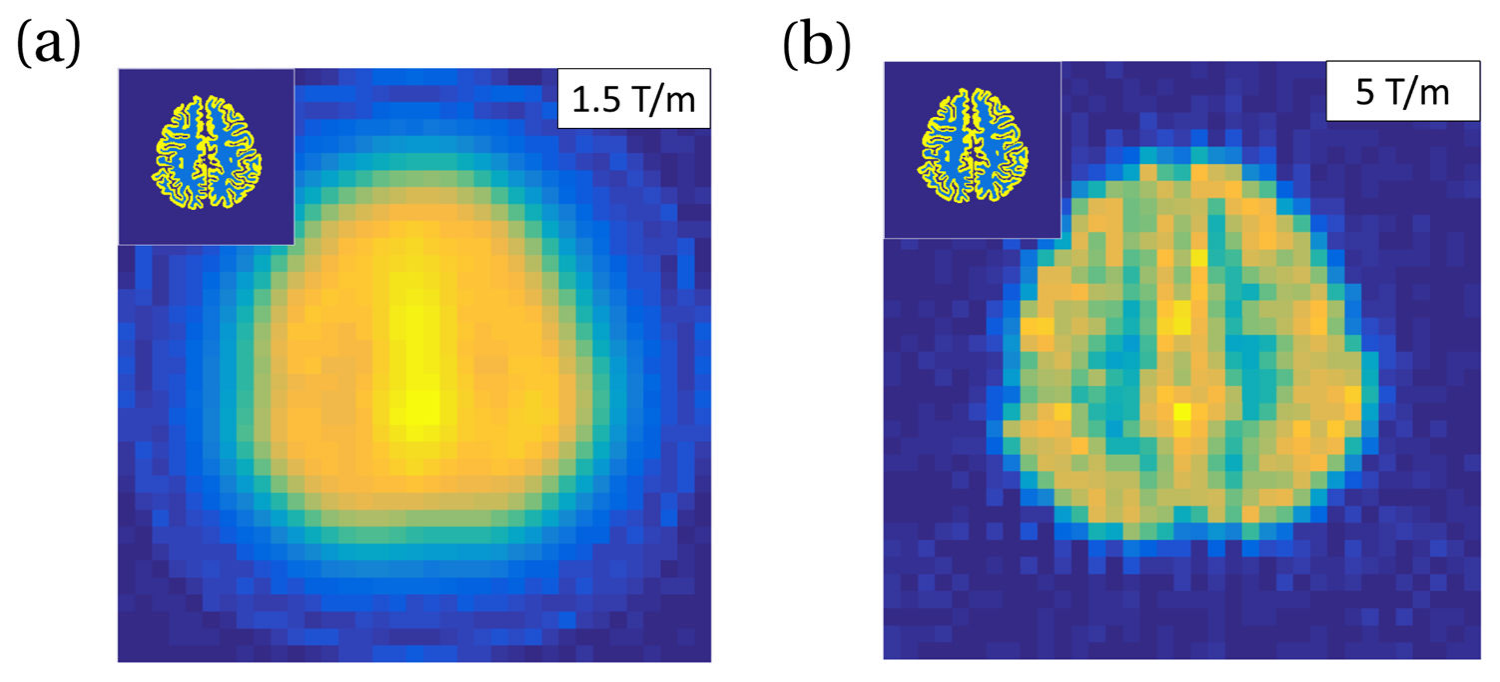
Simulated MPI projection FFL axial slice images of a FreeSurfer segmented human brain. Each image is a single frame, 3 sec scan time. Inset shows input brain segmentation of cortical gray matter with 4 ng Fe/mm^3^ (yellow in inset) and white matter with 0.8 ng Fe/mm3 (light blue in inset). PrecisionMRX^®^ SPION model used. Simulation scan parameters: 25 mT sinusoidal drive field at 10 kHz, human-sized solenoid receive coil with 25 turns (coil (c) in Tab. 1), signal filtered to 3rd harmonic. 9.77 nV noise added to represent the Nyquist noise in the receive coil at 100 kHz. 53 projection angles. Projection axis discretized with 35 points spanning 20 cm FOV. Gradient strength (a) 1.5 T/m, (b) 5 T/m.

Finally, we simulate an fMPI FFL projection brain image of blood volume contrast. We forma model object from a standard FreeSurfer segmented brain [34]. The model uses 400 mg Fe per 5 L of blood (5mg Fe/kg dose to 80 kg patient). Gray matter voxels are assigned 5% CBV and white matter voxels are assigned 1% CBV. This gives gray and white tissue iron concentrations of 4 ng Fe/mm^3^ and 0.8 ng Fe/mm^3^, respectively. The same human system scan parameters described above are used. The received signal is filtered to select only the 3rd harmonic component. Images are taken every 3 seconds. 200 images are averaged for a total imaging time of ~10 min, as described above. Fig. 12 shows the simulated images for a human system utilizing (a) 1.5 T/m gradient FFL and (b) 5 T/m gradient FFL, which would require improvements to the gradient field hardware.

## VII. Discussion and Conclusion

We have assessed the performance of projection FFL fMPI for the application to human functional neuroimaging. We focused on configurations that are achievable with conventional, readily available technologies. A gradient field of 1.5 T/m is achievable either with permanent magnets or electromagnets, and human-sized receive scanners can be sufficiently sensitive to detect the 20% changes in CBV caused by neural network activation with high CNR. While the human scanner is about 20× less sensitive than similar rodent scanners, it is still poised to detect the expected modulations of Fe in activated voxels with considerable CNR advantages over fMRI. Even further sensitivity increases could be achieved from improved SPIONs, cryogenic coils, and receive coil arrays. We intentionally focused our analysis on designs and technologies that are currently available. Further improvements in SPIONs and detection technologies will result in further advances in the sensitivity and resolution of fMPI.

We conclude that sensitivity is the relative strength of fMPI over other functional neuroimaging modalities, but given difficulties with human-sized FFL gradients and current SPION magnetization responses, spatial resolution is likely to be less of a gain. But although fMRI studies typically acquire with 2 mm or 3 mm spatial resolution and could be analyzed at this resolution, the data is usually spatially smoothed to about 6 mm resolution. Thus, the fMPI spatial resolution expected for human scanners (~7 mm) is similar to typical fMRI experiments. Without further refinements in hardware design and tracer optimization, the resolution and sensitivity of fMPI is very promising for human functional imaging. The expected improvements in sensitivity could help alleviate the need for averaging activation results over multiple subjects or for identifying subtle phenotypes or treatment responses in patient populations. Single subject measurements are, of course, mandatory for making diagnostic or disease phenotype statements in individuals. Beyond the increased sensitivity benefits, fMPI has additional advantages over fMRI. It is immune to the T2^*^ dropouts near the sinuses that plague MRI, as MPI can tolerate much higher field variation (1% vs. 10ppm for MRI). Additionally, since the baseline image signal level in fMPI comes only from the blood content of the voxel (5% of the voxel mass) and not (like MRI) from the water content (~100%), the expected percent changes on activation are larger (20%) rather than ~1% in fMRI. This helps reduce the effect of nuisance modulations of the signal, which appear as noise in the analysis of the activation time-series. In fMRI, these nuisance modulates are commonly referred to as “physiological noise” and are the dominant noise source in most fMRI studies. Because the percent signal change is ~20-fold higher for the fMPI contrast mechanism, we expect these sources will also be proportionally smaller. Provided robust and safe SPIONs, these improvements could potentially allow fMPI to replace fMRI in the same way that fMRI itself replaced O^15^ PET for the study of brain activation in the early 1990s.

## Figures and Tables

**Table 1:** Estimates of coil parameters and noise for various receive coil geometries (rodent-size (a)–(b), human head-size (c)–(h)). Estimates include: inductance, *z* component of *B*_1_ at center per unit Amp, body load loss (at 3rd harmonic frequency, 30 kHz) for cylindrical objects of conductivity 0.5 S/m ((a)–(b) cylinder: *d* = 4.5 cm, *l* = 4.5 cm, (c)–(h) cylinder: *d* = 22 cm, *l* = 22 cm), body load noise corresponding to this body load loss in 100 kHz BW, AC resistance in 4 parallel strands of 26 AWG Litz wire at 30 kHz, AC conducting noise corresponding to this AC resistance in 100 kHz BW, and relative detection sensitivity (*B*_1_ per unit Amp at center per AC conducting noise).

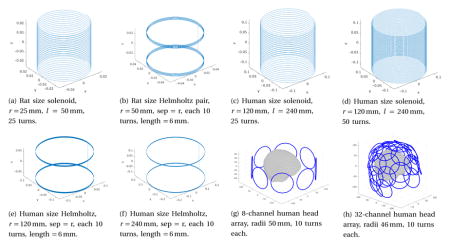							
Rx coil	L (*μ*H)	*z* component of *B*_1_ at center per unit Amp (*μ*T/A)	Body load loss 30 kHz (*μ*Ω)	Body load noise *σ* in 100 kHz BW(nV)	AC resistance @ 30 kHz (mΩ, per channel)	AC cond. Noise *σ* in 100 kHz BW (nV, channels combined)	Relative detect. sens. (*B*_1_ per A)/*σ* at center (*μ*T/A/nV)
**(a)**	19.81	443.70	0.008	0.11	13.2	4.46	99.5
**(b)**	51.11	166.77	0.001	0.04	21.1	5.64	29.6
**(c)**	133.30	93.65	0.963	1.20	63.3	9.77	9.6
**(d)**	533.20	185.16	3.850	2.41	126.6	13.81	13.4
**(e)**	151.02	72.65	0.593	0.95	50.7	8.74	8.3
**(f)**	344.95	36.90	0.153	0.48	101.3	12.36	3.0
**(g)**	161.90	0.336	–	–	10.6	11.31	0.03

## References

[1] Gleich B, Weizenecker J (2005). Tomographic imaging using the nonlinear response of magnetic particles. Nature.

[2] Goodwill PW, Lu K, Zheng B, Conolly SM (2012). An x-space magnetic particle imaging scanner. Rev Sci Instrum.

[3] Weber M, Erbe M, Bente K, Sattel TF, Buzug TM (2013). Scanner construction for a dynamic field free line in magnetic particle imaging. Biomed Tech.

[4] Bringout G, Graefe K, Buzug TM (2015). Performance and safety evaluation of a human sized ffl imager concept. International Workshop on Magnetic Particle Imaging.

[5] Borgert J, Schmidt JD, Schmale I, Bontus C, Gleich B, David B, Weizenecker J, Jockram J, Lauruschkat C, Mende O, Heinrich M, Halkola A, Bergmann J, Woywode O, Rahmer J (2013). Perspectives on clinical magnetic particle imaging. Biomed Tech/Biomed Eng.

[6] Mandeville JB, Marota JJ, Kosofsky BE, Keltner JR, Weissleder R, Rosen BR, Weisskoff RM (1998). Dynamic functional imaging of relative cerebral blood volume during rat forepaw stimulation. Magn Reson Med.

[7] Leite FP, Tsao D, Vanduffel W, Fize D, Sasaki Y, Wald LL, Dale AM, Orban GA, Rosen BR, Tootell RB, Mandeville JB (2002). Repeated fmri using iron oxide contrast agent in awake, behaving macaques at 3 tesla. Neuroimage.

[8] Christen T, Ni W, Qiu D, Schmiedeskamp H, Bammer R, Moseley M, Zaharchuk G (2013). High-resolution cerebral blood volume imaging in humans using the blood pool contrast agent ferumoxytol. Magn Reson Med.

[9] Qiu D, Zaharchuk G, Christen T, Ni WW, Moseley ME (2012). Contrast-enhanced functional blood volume imaging (ce-fbvi): Enhanced sensitivity for brain activation in humans using the ultrasmall superparamagnetic iron oxide agent ferumoxytol. Neuroimage.

[10] Gleich B (2014). Principles and Applications of Magnetic Particle Imaging.

[11] Zheng B, Vazin T, Goodwill PW, Conway A, Verma A, Saritas EU, Schaffer D, Conolly SM (2015). Magnetic particle imaging tracks the long-term fate of in vivo neural cell implants with high image contrast. Scientific Reports.

[12] Zheng B, von See MP, Yu E, Gunel B, Lu K, Vazin T, Schaffer DV, Goodwill PW, Conolly SM (2016). Quantitative magnetic particle imaging monitors the transplantation, biodistribution, and clearance of stem cells in vivo. Theranostics.

[13] Goodwill PW, Conolly SM (2010). The x-space formulation of the magnetic particle imaging process: One-dimensional signal, resolution, bandwidth, SNR, SAR, and magnetostimulation. IEEE Trans Med Imag.

[14] Goodwill PW, Konkle JJ, Zheng B, Saritas EU, Conolly SM (2012). Projection x-space magnetic particle imaging. IEEE Trans Med Imag.

[15] Knopp T, Sattel TF, Biederer S, Rahmer J, Weizenecker J, Gleich B, Borgert J, Buzug TM (2009). Model-based reconstruction for magnetic particle imaging. IEEE Trans Med Imag.

[16] Rahmer J, Weizenecker J, Gleich B, Borgert J (2012). Analysis of a 3-d system function measured for magnetic particle imaging. IEEE Trans Med Imag.

[17] Konkle JJ, Goodwill PW, Carrasco-Zevallos OM, Conolly SM (2012). Projection reconstruction magnetic particle imaging. IEEE Trans Med Imag.

[18] Konkle JJ, Goodwill PW, Saritas EU, Zheng B, Lu K, Conolly SM (2013). Twenty-fold acceleration of 3d projection reconstruction mpi. Biomed Tech/Biomed Eng.

[19] GMW Associates Gmw electromagnet coils.

[20] Pourrahimi S, Pourrahimi N, Punchard WF, Starewicz PM High magnetic field gradient strength superconducting coil system.

[21] Lövsund P, Oberg PA, Nilsson SE (1980). Magneto- and electrophosphenes: a comparative study. Med Biol Eng Comput.

[22] Goodwill PW, Conolly SM (2011). Multidimensional x-space magnetic particle imaging. IEEE Transactions on Medical Imaging.

[23] Haegele J, Rahmer J, Gleich B, Borgert J, Wojtczyk H, Panagiotopoulos N, Buzug TM, Barkhausen J, Vogt FM (2012). Magnetic particle imaging: Visualization of instruments for cardiovascular intervention. Radiology.

[24] Cauley S A parallel imaging reconstruction to allow partial fov projections in mpi.

[25] Saritas EU, Goodwill PW, Zhang GZ, Conolly SM (2013). Magnetostimulation limits in magnetic particle imaging. IEEE Trans Med Imag.

[26] Bozkurt E, Demirel OB, Sarica D, Muslu Y, Saritas EU Effects of safety limits on image quality in mpi.

[27] Panagiotopoulos N, Duschka RL, Ahlborg M, Bringout G, Debbeler C, Graeser M, Kaethner C, Lüdtke-Buzug K, Medimagh H, Stelzner J, Buzug TM, Barkhausen J, Vogt FM, Haegele J (2015). Magnetic particle imaging: current developments and future directions. Int Journ Nanomed.

[28] Bauer LM, Situ SF, Griswold MA, Samia ACS (2015). Magnetic particle imaging tracers: State-of-the-art and future directions. J Phys Chem Lett.

[29] Myers W, Slitcher D, Hatridge M, Busch S, Mössle M, McDermott R, Trabesinger A, Clarke J (2007). Calculated signal-to-noise ratio of mri detected with squids and faraday detectors in fields from 10 microt to 1.5 t. J Magn Reson.

[30] Darrasse L, Ginefri JC (2003). Perspectives with cryogenic rf probes in biomedical mri. Biochimie.

[31] Gunel B, Zheng B, Conolly SM An ultra-low noise preamplifier design for magnetic particle imaging.

[32] Krestel E Imaging Systems for Medical Diagnosis: Fundamentals and Technical Solutions - X-ray Diagnostics - Computed Tomography - Nuclear Medical Diagnostics -Magnetic Resonance Imaging - Ultrasound Technology.

[33] New England Wire Technologies LitzWire Theory.

[34] Fischl B, Salat DH, Busa E, Albert M, Dieterich M, Haselgrove C, van der Kouwe A, Killiany R, Kennedy D, Klaveness S, Montillo A, Makris N, Rosen B, Dale AM (2002). Whole brain segmentation: Automated labeling of neuroanatomical structures in the human brain. Neuron.

